# Plasma proteome analysis of patients with type 1 diabetes with diabetic nephropathy

**DOI:** 10.1186/1477-5956-8-4

**Published:** 2010-02-03

**Authors:** Anne Julie Overgaard, Henning Gram Hansen, Maria Lajer, Lykke Pedersen, Lise Tarnow, Peter Rossing, James N McGuire, Flemming Pociot

**Affiliations:** 1 Department of Genome Biology, Hagedorn Research Institute, Gentofte, Denmark; 2 Department of Research in Complications, Steno Diabetes Center, Gentofte, Denmark; 3 Center for Models of Life, Niels Bohr Institute, Copenhagen, Denmark

## Abstract

**Background:**

As part of a clinical proteomics program focused on diabetes and its complications we are looking for new and better protein biomarkers for diabetic nephropathy. The search for new and better biomarkers for diabetic nephropathy has, with a few exceptions, previously focused on either hypothesis-driven studies or urinary based investigations. To date only two studies have investigated the proteome of blood in search for new biomarkers, and these studies were conducted in sera from patients with type 2 diabetes. This is the first reported in depth proteomic study where plasma from type 1 diabetic patients was investigated with the goal of finding improved candidate biomarkers to predict diabetic nephropathy. In order to reach lower concentration proteins in plasma a pre-fractionation step, either hexapeptide bead-based libraries or anion exchange chromatography, was performed prior to surface enhanced laser desorption/ionization time-of-flight mass spectrometry analysis.

**Results:**

Proteomic analysis of plasma from a cross-sectional cohort of 123 type 1 diabetic patients previously diagnosed as normoalbuminuric, microalbuminuric or macroalbuminuric, gave rise to 290 peaks clusters of which 16 were selected as the most promising biomarker candidates based on statistical performance, including independent component analysis. Four of the peaks that were discovered have been identified as transthyretin, apolipoprotein A1, apolipoprotein C1 and cystatin C. Several yet unidentified proteins discovered by this novel approach appear to have more potential as biomarkers for diabetic nephropathy.

**Conclusion:**

These results demonstrate the capacity of proteomic analysis of plasma, by confirming the presence of known biomarkers as well as revealing new biomarkers for diabetic nephropathy in plasma in type 1 diabetic patients.

## Background

Diabetic nephropathy will affect approximately 30% of all patients with diabetes [1,2]. The proportion of patients that progress to end stage renal disease (ESRD) because of diabetic nephropathy has been estimated to be 7% [3] and as a consequence diabetic nephropathy is the most common cause of renal failure in the developed world [4,5]. Diabetic nephropathy advances through a number of recognizable steps from sub-clinical disease to the first measurable stage of microalbuminuria (MIC), defined as persistent albumin excretion levels in urine normalized to creatinine levels (U-albumin) of 30-300 mg/24 h, to macroalbuminuria/diabetic nephropathy (DMN) with U-albumin>300 mg/24 h. DMN is followed by renal dysfunction and ultimately ESRD. Although positive effects on the development and progression of diabetic nephropathy through strict control of blood glucose [6], blood pressure [7] and in particular blockade of the renin-angiotensin system [8,9] have been reported, it still has not been enough to prevent the high incidence of end stage kidney damage caused by diabetes. Administration of cardiovascular drugs, which are commonly prescribed for patients with MIC or DMN, can markedly decrease the urinary albumin excretion rate (UAER) without concomitant improvement of the disease state.

In order for intervention to have optimal effect on prevention of ESRD, initiation early on in the disease process is crucial. At present MIC is used as the best risk marker for development of diabetic nephropathy; however, the number of patients with MIC that progress to DMN is less in recent studies compared to previously, and some even regress to normoalbuminuria (N)(U-albumin<30 mg/24 h) [1].

The search for new biomarkers for diabetic nephropathy, with a few exceptions, has focused on either hypothesis-driven studies or urinary-based proteomics [10-13]. To date only two studies have investigated the proteome of blood in search for biomarkers and these studies were accomplished with sera from patients with type 2 diabetes (T2D) [14,15]. This study focuses on analyzing plasma from type 1 diabetic (T1D) patients because of its advantage in reflecting more general changes that occur in the human body and because it is a relatively stable biological fluid that does not require normalization, as is the case with urine.

The main challenge in plasma proteome research is that candidate biomarkers are present in trace amounts among a large background of non-relevant and abundant proteins. A multitude of pre-fractionation techniques has been described [16], but the majority of them are inherently low throughput and are not compatible with the analysis of individual patient samples. Bead-based fractionation methods, on the other hand, offer a workflow that is amenable to automation and clinical proteomics workflows. For the present study, two different bead-based techniques were chosen for biomarker discovery: anion exchange and hexapeptide library resins. Anion exchange is used to separate proteins in plasma according to their isoelectric points, whereas the hexapeptide library beads can drastically reduce the amounts of the most abundant proteins in plasma while simultaneously enhancing the concentration of the most dilute species [17]. The techniques were combined with surface enhanced laser desorption/ionization time-of-flight mass spectrometry (SELDI-TOF-MS) and independent component analysis (ICA) to detect changes in protein levels between patients with N, MIC and DMN. Earlier discovery of patients most at risk for developing diabetic nephropathy could allow early initiation of intervention and a more tailored management of late complications in diabetes.

## Results

### Clinical data

Patient groups in the cross-sectional cohort were matched with respect to gender, duration of diabetes and body mass index (BMI) but differed slightly by age (p = 0.01). The DMN group had significantly lower estimated glomerular filtration rates (eGFR) compared to the other groups (p < 0.0001) while equivalent eGFR values were observed in the N and MIC groups. There were no significant differences in systolic blood pressure or diastolic blood pressure for comparisons of all groups or for levels of serum cholesterol between the groups. Haemoglobin A1c (HbA1c) differed significantly for an all group comparison (p = 0.02), this was caused by a significant difference between the N and DMN groups. The clinical data are summarized in Table 1.

**Table 1:** Clinical data of the diabetic patients differentiated according to level of albuminuria

	N	MIC	DMN	p value
n, total	42	40	41	
Gender, Male (female)	19 (23)	18 (22)	22 (19)	0.25
Age (years)	56 (11)	55 (11)	49 (10)*	0.01
DM duration (years)	36 (11)	36 (11)	34 (10)	0.58
BMI (kg/m^2^)	24.5 (2.8)	25.2 (3.7)	25.3 (4.5)	0.62
HbA1c (%)	8.2 (1)	8.8 (1.2)	8.9 (1.1)*	0.02
Creatinine (μmol/l)^◆^	91 (82-96)	90 (81-102)	124 (99-172)* ^,§^	<0.0001
U-albumin (mg/g)^◆¶^	6 (4-8)	23 (9-59)	453 (195-936) * ^,§^	<0.0001
eGFR(ml/min/1.73 m^2^)	70.1 (10.7)	68.6 (11.4)	49.5 (18.5) * ^,§^	<0.0001
Cholesterol (mmol/l)	4.8 (0.8)	5 (1)	4.9 (1)	0.55
Systolic BP (mmHg)	138 (23)	140 (23)	144 (19)	0.42
Diastolic BP (mmHg)	74 (10)	73 (12)	78 (10)	0.09

Numbers are presented as mean (SD). ◆ Numbers are presented as median (IQR). ¶ Some patients had U-albumin levels reduced by antihypertensive medication which was not stopped when spot urine samples were collected for the study.

* Indicates that means differed significantly between DMN group and N group.

§ Indicates that means differed significantly between DMN group and MIC group.

# Indicates that means differed significantly between MIC group and N group.

n, numbers; DM, diabetes mellitus; HbA1c, hemoglobin A1c; U-albumin, urinary albumin levels normalized to creatinine levels; eGFR, estimated glomerular filtration rate; BP, blood pressure

### SELDI-TOF-MS

Plasma complexity was reduced prior to proteomic analysis by anion exchange or hexapeptide fractionation. Analysis of individual T1D patients resulted in more than 1000 protein and peptide peaks in the spectra generated by SELDI-TOF-MS. After spectral processing and cluster selection, the data for individual peaks were subjected to stringent quality control as a part of feature reduction, which resulted in a total of 518 peak clusters (179 from anion exchange and 339 from hexapeptide studies). The peak data were further reduced prior to final data analysis to exclude all peaks that were highly correlated to avoid discovering redundant candidate biomarkers. The final peak lists included 290 peak clusters (111 from anion exchange and 179 from hexapeptide studies).

### ICA

One of the challenges in SELDI-TOF-MS profiling, or other top-down approaches such as matrix assisted laser desorption time-of-flight mass spectrometry (MALDI-TOF-MS), is the reduction of false positives that arise from the nature of the data. Spectra acquired by SELDI-TOF-MS are complex because they are composed of a number of peaks, some of which are highly correlated, that are polluted by noise as well as artefacts of biological, chemical or physical origin. One potential solution to this problem is the application of ICA, which is a signal processing technique used to separate distinct underlying signals from mixed recorded signals. The observed signals are the protein peak clusters, believed to be independent of each other, and can be characterised as comprised of a mixture of the independent components (ICs). Components can be evaluated on the basis of their statistical properties [18]. The ICs that were capable of separating the groups based on a Kruskal-Wallis (KW) test were selected and the peaks that contributed significantly to the IC were identified by having a high absolute load value in the component matrix. Each component was further tested for its stability by multiple runs of the algorithm where the mean of the correlation between the components in the component matrix was used as a stability score of the component. This was done to ensure robustness and to avoid finding components defining local maxima, such as noise or artefacts. All p values were adjusted for multiple testing by the Benjamini-Hochberg algorithm [19]. Selection of proteins for future research were based on two different criteria: either proteins contributing to components with stability >0.8 and KW p value < 0.0001 or peaks differing significantly between groups with a KW p value < 0.0001. Tables 2 and 3 list the 16 peaks selected for future investigation.

**Table 2:** Protein peaks contributing significantly to top ICA components.

Component	Stability Score	p value	Peak(s) (m/z)	Protein Identity(ies)
5	0.809212	2.67E-07	13880	Transthyretin*
59	0.801315	3.62E-06	11351 13880	- Transthyretin*
15	0.85689	9.79E-06	6963.9	-
91	0.807664	3.43E-05	28121 6631.5	Apo A1* Apo C1**
28	0.807874	4.93E-05	17278	-
34	0.877577	6.02E-05	28111	Apo A1*
95	0.823827	9.07E-05	5684.4 28111	- Apo A1*
116	0.822404	9.07E-05	8938.3	-
33	0.857141	9.07E-05	17396	-

*Proteins identified by sequencing. **Proteins identified by immunoprecipitation, sequencing and westernblots. m/z, mass to charge. P values are Benjamini-Hochberg corrected for multiple testing.

**Table 3:** Proteins selected based on the lowest KW value in comparison of the three groups.

p value	Peak (m/z)	Protein Identity
1.12E-06	13584	-
3.13E-06	9101.8	-
3.13-06	28121	Apo A1*
3.64E-06	13350	Cystatin C**
3.64E-06	2956.7	-
9.42E-06	13880	Transthyretin*
1.51E-05	11344	-
6.99E-05	6963.9	-
7.34E-05	9185.4	-
9.73E-05	13797	Transthyretin*

*Proteins identified by sequencing. **Proteins identified by immunoprecipitation. P values are Benjamini-Hochberg corrected for multiple testing.

### Identification of candidate biomarkers

**Figure 1:**
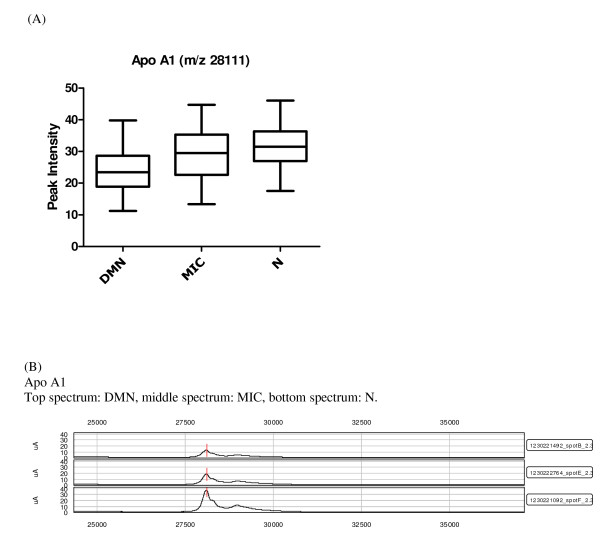
**Candidate biomarkers for diabetic nephropathy**. (A) Box and whiskers plot of apo A1 intensity, where the three diabetic groups are divided by albuminuria, the ordinate represents the peak intensity for the marker. (B) SELDI-TOF-MS spectra of known markers of diabetic nephropathy significantly different between the groups: apo A1, the ordinate is the m/z of the peak, and the abscissa is the relative intensity.

**Figure 2:**
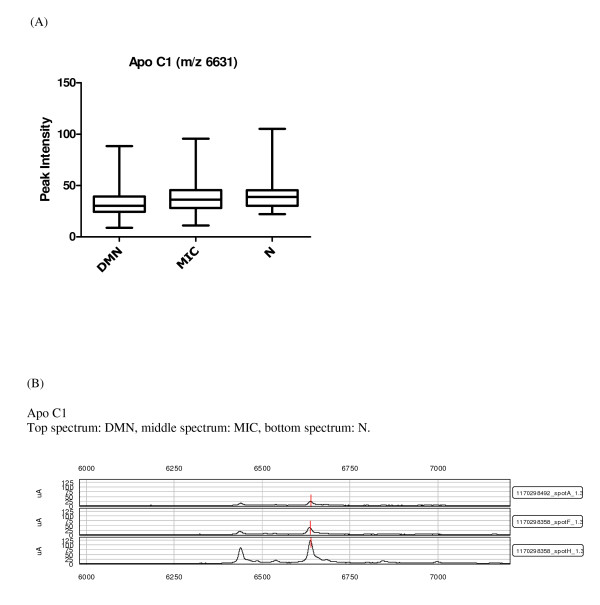
**Candidate biomarkers for diabetic nephropathy**. (A) Box and whiskers plot of apo C1 intensity, where the three diabetic groups are divided by albuminuria, the ordinate represents the peak intensity for the marker. (B) SELDI-TOF-MS spectra of known markers of diabetic nephropathy significantly different between the groups: apo C1, the ordinate is the m/z of the peak, and the abscissa is the relative intensity.

**Figure 3:**
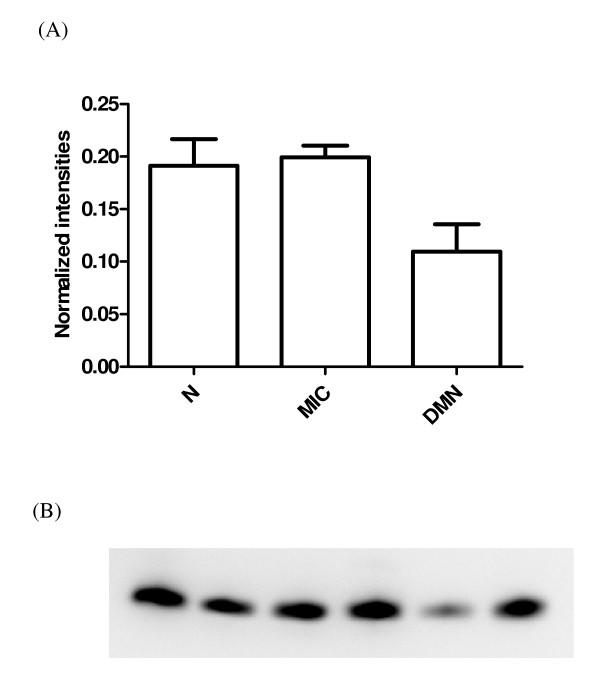
**Western blots of apo C1**. (A) The chart represents the normalized average intensities (SD) of two independent gels of the same six samples, two from each group of diabetic patients. The bands intensities where detected by the computer software Multi Gauge version 2.0 from Fujifilm. (B) On the gel the first two lanes, from left to right, is two samples from the N group, next two lanes are two samples from the MIC group, and the last two are from the DMN group.

**Figure 4:**
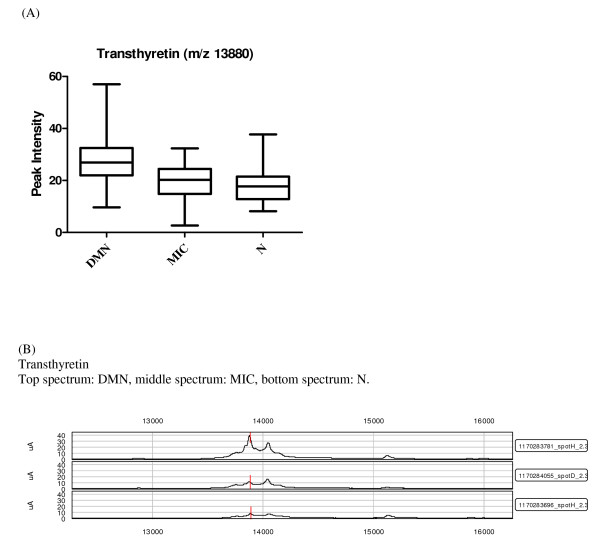
**Candidate biomarkers for diabetic nephropathy**. (A) Box and whiskers plot of transthyretin intensity, where the three diabetic groups are divided by albuminuria, the ordinate represents the peak intensity for the marker. (B) SELDI-TOF-MS spectra of known markers of diabetic nephropathy significantly different between the groups: transthyretin, the ordinate is the m/z of the peak, and the abscissa is the relative intensity.

**Figure 5:**
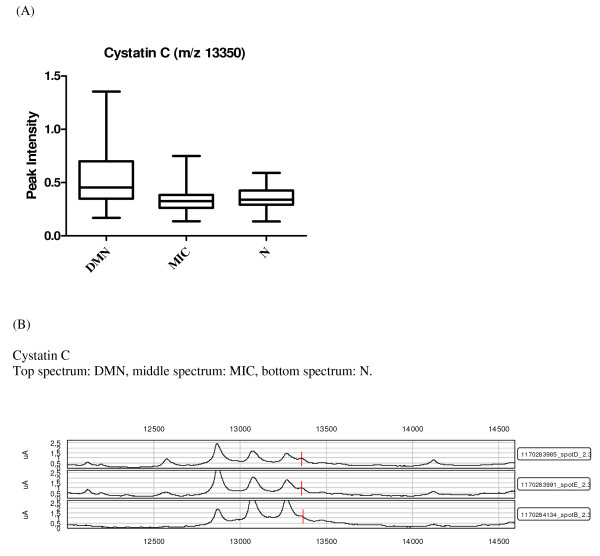
**Candidate biomarkers for diabetic nephropathy**. (A) Box and whiskers plot of cystatin C intensity, where the three diabetic groups are divided by albuminuria, the ordinate represents the peak intensity for the marker. (B) SELDI-TOF-MS spectra of known markers of diabetic nephropathy significantly different between the groups: cystatin C, the ordinate is the m/z of the peak, and the abscissa is the relative intensity.

Overlap between the two data sets from each fractionation method is a possibility, but cannot be determined directly without protein identification, which is not an integral part of a SELDI-TOF-MS study and can be extremely time consuming. The two data sets are therefore considered as independent of one another. Only previously established or particularly promising candidates are identified at this stage of biomarker discovery. Among the highest contributing proteins in the chosen components and peaks in diabetic nephropathy, impaired renal function and other diseases were the following: transthyretin, apolipoprotein C1 (apo C1), apolipoprotein A1 (apo A1) and cystatin C. The identities of these proteins were confirmed by immunoprecipitation and sequencing or western blots. Apo A1 (28111/28121 Da) was observed in three of the components and was also on the list of proteins chosen based on the p values from the KW analysis (Table 2 and 3), where the lowest levels of the protein were found in the DMN group (Figure 1). Apo C1 (6631.5 Da), a protein related to diabetes and diabetic nephropathy was identified as contributing significantly, along with apo A1, in component 91 (Table 2). Furthermore a KW analysis of the difference in peak intensities between the groups showed that the means differed significantly between all groups (p = 0.012), with the lowest levels found in the DMN group (Figure 2). These results were confirmed with western blotting that verified lower apo C1 levels in the DMN group compared to the MIC and N groups (Figure 3). The expression levels of transthyretin (13797/13880 Da) and cystatin C (13350 Da), two established diabetes or renal function-related related proteins, were significantly lower in the DMN group compared to the other groups (p < 0.0001) but equivalent transthyretin and cystatin C levels were observed in the N and MIC groups (Figure 4 and 5 respectively).

### Reproducibility

The reproducibility of all analyses was followed using reference standards subjected to the same workflow as patient samples. The inter-assay coefficients of variation (CV) was 23.4% (15.4%-29%) for CM10 arrays and 20.8% (15.2%-28.6%) for Q10 arrays after hexapeptide fractionation, whereas the CV after anion exchange fractionation was 21.8% (19.1%-27%) for CM10 arrays and 23.7% (19.7%-29.1%) for IMAC30 arrays. Additional file 1; Table S1 describes the main differences and results obtained with the two different fractionation strategies.

## Discussion

The current best predictive marker of future development of diabetic nephropathy is MIC in both T1D and T2D. The development of renal disease from the first clinical sign of MIC to DMN is characterised by progressively damaged glomerular capillary wall function and breakdown of the filtration barrier. The stage of MIC already represents a measurable level of diabetic kidney disease where advanced structural renal damage has occurred and may progress further independent of metabolic control [20,21]. Although the measurement of albuminuria is currently the best available non-invasive method for early detection of pending renal disease in diabetic patients, much debate exists in the current literature about its sensitivity and specificity [1]. Urinary albumin content is influenced by several factors such as intensive physical activity, the menstrual cycle, infection of the urinary system, hypertension and other nephropathies [12]. Consequently, new and better biomarkers and risk predictors are needed.

Lower levels of apo C1 were found in the micro- and macroalbuminuric groups compared to normoalbuminuric, which differs from what is previously reported [22]. Hirano *et al *found that apo C1 levels in the very low-density lipoproteins (VLDL) increased with the growing severity of diabetic nephropathy. A possible explanation could be that our data reflects the total content of apo C1 in both HDL and VLDL particles in the plasma whereas Hirano *et al *measured apo C1 incorporated in the VLDL particles only.

Furthermore, apo A1, cystatin C and transthyretin levels were all identified as either contributing significantly to components and/or were significantly different between the groups based on a KW analysis, this is in accordance to what has previously been described about these proteins, and it has been proposed that these proteins could have diagnostic value in DMN [23-26]. The candidate biomarkers discovered in this cross-sectional cohort may turn out to be progression biomarkers, but they need to be corroborated in a longitudinal cohort. The nature of the current cohort, in which the DMN group had varying degrees of duration, resulted in a spread in U-albumin values at the time of blood sampling due to antihypertensive treatment of the DMN and MIC patients. The presence of a standard treatment in the cohort brings robustness and persistence to the actually obtained candidate biomarkers and reflects the everyday clinical setting.

Interestingly, a number of not yet described protein peaks were identified in this study, probably as a result of the initial plasma complexity reduction prior to the SELDI-TOF-MS analysis. The intensity of these candidate markers were very different between the groups and could have future potential in contributing to a superior model of proteins that are able to predict nephropathy in diabetic patients. At present we are working on establishing the identity of these proteins.

## Conclusion

In this study we evaluated whether it is possible to distinguish plasma protein profiles from T1D patients with various degrees of albuminuria using SELDI-TOF-MS and bioinformatic tools. ICA identified 16 candidate peaks that contributed significantly in their respective components with high stability and ability to separate the groups. ICA has previously been applied to extract reliable protein signals from MALDI-TOF-MS spectra, where the algorithm also was used to detect differences in protein peaks between experimental groups [27]. The technique has the potential to considerably increase the quality of the resulting data and improve the biological validity of subsequent examination and seems to be a promising tool for biomarker discovery studies. Future research is aimed at analysing larger groups of samples. After establishing a model based on proteomic patterns for the separation of the three groups, the model will be validated in longitudinal sample sets to determine the diagnostic and predictive value of the obtained protein profiles. It is our goal to find biomarkers able to predict, detect and monitor the progression of DMN and the effect of reno- protective intervention in diabetic patients.

## Materials and methods

### The Cohort

The participants consisted of Caucasian patients with T1D examined at Steno Diabetes Center in 2004 and the samples chosen for the current study were divided into three groups based on albumin levels in 24 hour urine collections analysed as part of the routine care: 42 with N (U-albumin < 30 mg/24 h), 40 with MIC (at least two out of three consecutive urines with albumin excretion rate 30-300 mg/24 h) and 41 patients with persistent DMN (U-albumin > 300 mg/24 h). The interval between DMN diagnosis and sample collection of the cohort was variable. The DMN group was selected for the previously diagnosed presence of diabetic nephropathy independent of its duration. The majority of the group was being treated to reduce their blood pressure and subsequently, their U-albumin at the time of sampling was not necessarily representative of disease progression. At the time of plasma sampling, 26 out of 41 patients still had an U-albumin of > 300 mg/day while 13 had intermediate levels and only two were lowered enough to be placed in the N group if they had not previously been diagnosed. Of the 15 patients whose levels were lower than at the point of diagnosis, five were put on a short pause from their antihypertensive medication and three of them returned to U-albumin levels above 300 mg/day before resuming their medication. The two other patients had level increases up to 206 and 291 mg/day, values which approach the cutoff for DMN, and support the original diagnosis. In a follow-up period four years after sampling there were only three patients (two from MIC and one from N) who had progressed to diabetic nephropathy.

Originally, groups were matched by gender, age (± 5 years) and duration of diabetes (± 3 years) (>20 years, for normoalbuminuric patients). Investigations were performed in the morning after an overnight fast. Arterial blood pressure was measured three times with an appropriate cuff size following at least 10 min supine rest. Urinary albumin concentration was measured by an enzyme immunoassay from early morning spot urine collections. Serum and urine creatinine concentration was assessed by a kinetic Jaffé method [28]. Glomerular filtration rate was estimated (eGFR) using the 4 variable MDRD GFR formula (age, gender, race, serum creatinine) http://mdrd.com/. Plasma samples were stored at -80 C until analysis. The study was approved by the local ethics committee and all patients gave their informed consent.

Two samples from DMN, two from MIC and one from N did not contain the adequate volume for the hexapeptide fractionation and where left out as compared to the anion exchange method.

### Plasma Fractionation

The patient plasma samples along with aliquots of reference human serum which we used to measure reproducibility, were fractioned in a high through put automated system by two different techniques: anion exchange chromatographic Q Ceramic HyperD 20 resin (Pall, East Hills, New York, USA) and with ProteoMiner hexapeptide library beads (BioRad, Malvern, Pennsylvania, USA). For the anion exchange chromatography fractionation bulk resin was washed and equilibrated with 50 mM Tris (pH 9) and adjusted to a 50% slurry. Aliquots of the resin were dispensed into wells of filter plates (Nunc, Rochester, New York, USA) and washed twice with 50 mM Tris (pH 9). Plasma samples from each patient were centrifuged for 20 min at 10,000 × g and an aliquot (50 μL) of each was partially denatured by mixing with 50 μL 9 M urea + 2% CHAPS in 100 mM HEPES (pH 7) for 30 min at 4°C. The plasma samples were transferred to the equilibrated resin in the filter plates and incubated with shaking for 30 min at 4°C. The flow through (Q FT) was collected along with a 100 μL 100 mM HEPES (pH 7.0) + 0.01% OGP wash. A series of step-wise elutions (2 × 100 μL) was performed: 100 mM HEPES (pH 7.0) + 0.01% OGP (Q E1), 100 mM Sodium Acetate (pH 5.0) + 0.1% OGP (Q E2), 100 mM Sodium Acetate (pH 4.0) + 0.1% OGP (Q E3) and 3.0 M Guanidine (in 100 mM Sodium Acetate (pH 4.0) + 0.1% OGP) (Q E4). The resin was washed with 25/25/50 Acetonitrile/2-propanol/Water + 0.1% TFA (2 × 100 μL, Q E5).

The fractionation of plasma with hexapeptide beads was performed using ProteoMiner beads. Plasma was incubated with the resin as described previously and proteins where eluted sequentially with the four following solutions: 1 M sodium chloride in 20 mM HEPES (pH 7.0) (PM F1), 0.2 M Glycine (pH 2.4) (PM F2), 60% (w/v) ethylene glycol in water (PM F3) and 33.3% (v/v) 2-propanol, 16.7% (v/v) acetonitrile, 0.1% (v/v) triflouroacetic acid (PM F4).

### Preparation of SELDI-TOF-MS Arrays

The plasma fractions from both preparation techniques, with the exception of Q E5 and the flow through (PM FT) from the ProteoMiner preparation were analyzed by SELDI-TOF-MS. The fractions from the anion exchange preparation were analyzed on cation exchange (CM10), and in some cases, immobilized metal affinity (IMAC30) arrays. The ProteoMiner fractions were all analyzed on CM10 arrays with the addition of strong anion exchange (Q10) arrays on PM F2 and PM F3. The IMAC30 arrays were charged with copper (Q E1) or nickel (Q FT, Q E2 and Q E3). The arrays were prepared as described previously [29,30]. Sinapinic acid was used as the matrix for all array preparations. The arrays were prepared using an automated system and read in a PCS-4000 instrument (Bio-Rad, Malvern, Pennsylvania, USA) with a high and a low laser setting. Detailed protocols on data acquisition and data processing are listed as supplementary information in additional file 2.

### Statistical Analysis

The clinical data was evaluated using the GraphPad Prism statistical software. All group comparisons where done by a one-way ANOVA followed by a Tukey post-test, and the individual group comparisons by a student's t-test. For non-ordinal data, a KW test followed by a Dunns post-test, where used for all group comparison and a Mann-Whitney test for evaluation of the individual groups in between.

Statistical calculations of the proteomics data were performed using the R software environment http://www.r-project.org. The analysis of the data from the anion exchange fractionation was done in a two stage process. In the first stage all two group comparisons were performed using the student's t-test. The three diabetic groups were analyzed using an analysis of variance (ANOVA) p value derived from a linear model. Two comparisons were employed for feature reduction: p values derived from the comparison of the N/DMN groups and the ANOVA for all three diabetic groups. All peak clusters with a p value of ≤ 0.01 (223 for the student's t-test and 187 for the ANOVA test) were considered for further analysis. The t-test had a statistical power of 0.97 (n = 42 (per group), α = 0.01) while the ANOVA had a statistical power of 0.99 (n = 42 (per group), α = 0.01, between group variance = 1, within group variance = 3).

A more precise cluster alignment was achieved by performing an internal mass calibration using the average mass for a cluster for which a peak appeared in every single spectrum. This method allowed for an improved alignment using peaks that are common to all spectra and does not rely on previous knowledge of a peak's identity. The peaks selected in the first stage were manually relabeled in the aligned spectra. All peaks not deemed to be true peaks were excluded from further analysis. A total of 179 peak clusters were carried forward.

The data structure complexity and redundancy was reduced by identifying peak families (Spearman correlation coefficient ≥ 0.85) and retaining only the most intense member. Both a KW test with a Benjamini-Hochberg correction for multiple testing and ICA were performed on the remaining 111 peak clusters. ICA was performed using 100 components and run a total of five times. The results from each run were tested for stability by comparing the results with a set of 100 independent runs. Only components with a stability score (min. - max., 0 - 1.0) of ≥ 0.80 were retained. The performance of each component was tested using a KW test with a Benjamini-Hochberg correction for multiple testing.

The spectra obtained from the hexapeptide fractionation of plasma were initially internally calibrated by cluster alignment as mentioned above before statistical analysis in a two stage process. In the first stage all two group comparisons were performed using a Mann-Whitney U test, and the three diabetic groups were additionally compared using a KW test. Two conditions were employed for feature reduction: p values ≤ 0.05 derived from the comparison of the N/MA groups and p values ≤ 0.05 acquired from the comparison of the three diabetic groups by the KW test.

Based on the data from the Q fractionation, we estimate that at 95% power at a significance level of 0.05 group differences will be significant assuming a within group variance of 0.69. The comparisons can detect differences between groups using 28 or more participants.

In the second stage all peak clusters selected were manually relabeled and all peaks not judged to be true peaks were excluded from further analysis. A total of 339 peak clusters were retained. The data was analyzed identically as for the anion exchange fractionation with the difference that each of the fractions from the hexapeptide bead treatment were considered independent of one another and were carried forward separately (179 peak clusters). ICA was performed using 117 components.

### Immunoprecipitation

The identities of cystatin C, transthyretin, apo C1 and apo A1 were confirmed using Dynabeads protein-G beads (Invitrogen, Carlsbad, California, USA) and specific antibodies (Dako, Carpinteria, California, USA or Abcam, Cambridge, Massachusetts, USA). The unbound sample and the eluted protein was analyzed on SELDI-TOF-MS to confirm depletion elution of the right peak. Additionally, the eluted protein was trypsin digested and analyzed on ESI ion trap MS. Detailed protocols for the immunoprecipitation and trypsin digestion are listed in the supplementary information in additional file 2. The identities of the digested proteins were identified based on matching the MS/MS data with mass values calculated for selected ion series of a peptide. A protein database was searched without applying any constraints on molecular weight or species by Mascot (Matrix Science Inc., Boston, Massachusetts, USA) [31]. Transthyretin and apo A1 were identified with several peptide matches, but apo C1 was identified on the basis of a single peptide. The amino acid sequences were blasted and identified by Mascot and the identification approved if the score of the peptides where equal or exceeded the score reported by mascot to be significant as previously described [31].

### Detection and quantification of Apo C1 by Western blots

As prove of concept of the semi quantitative abilities of SELDI-TOF-MS and to confirm the identity of apo C1 we performed western blots. Protocols for western blots are listed in supplementary information in additional file 2.

Supplementary information is available at Proteome Science's website.

## Competing interests

The authors declare that they have no competing interests.

## Authors' contributions

AJO carried out the SELDI-TOF-MS analysis, the immunoprecipitation, the sequencing, the statistical analysis and drafted the manuscript. HGH participated in the western blots and the interpretation of data. ML participated in the collection of the clinical samples and interpretation of the data. LP participated in the independent component analysis. LT participated in the design and coordination of the study, and measured the clinical parameters of the patients in the cohort. PR participated in the design and coordination of the study and helped to draft the manuscript. JNM helped with all the technical and statistical analysis and participated in the design of the study and interpretation of the results and the drafting of the manuscript. FP participated in the design of the study and interpretation of the data and writing of the manuscript. All authors read and approved the final manuscript.

## Supplementary Material

Additional file 1 **Supplementary Table**. SELDI-TOF-MS analysis of the two different fractionation techniques. Click here for file

Additional file 2 **Supplementary information**. Detailed protocols of data acquisition and processing, immunoprecipitation, trypsin digestion and western blotting. Click here for file
