# Supplementary material for: Plasma proteome analysis of patients with type 1 diabetes with diabetic nephropathy
Source: Proteome Sci. 2010 Feb 3;8:4. doi: 10.1186/1477-5956-8-4 (PMC2827395; doi:10.1186/1477-5956-8-4)
Supplement: Additional file 1 — Supplementary Table. SELDI-TOF-MS analysis of the two different fractionation techniques. [file 1477-5956-8-4-S1.DOC]

**Table S1 - SELDI-TOF-MS analysis of the two different fractionation techniques**

|  | | ProteoMiner fractionation | Q fractionation |
| --- | --- | --- | --- |
| Start material | | 1000 µL plasma | 50 µL plasma |
| Flow through | SELDI array surfaces | - | CM10 & IMAC30- Ni |
|  | Average CV (%) | - | 25.25 |
|  | No. candidate biomarkers | - | 49 |
| Elution 1 | Buffer | High salt, pH 7.0 | pH 7.0 |
|  | SELDI array surfaces | CM10 | IMAC30-Ni |
|  | Average CV (%) | 27.66 | 25.59 |
|  | No. candidate biomarkers | 12 | 5 |
| Elution 2 | Buffer | Acidic (pH 2.4) | pH 5.0 |
|  | SELDI array surfaces | CM10 & Q10 | - |
|  | Average CV (%) | 25.64 | - |
|  | No. candidate biomarkers | 58 | - |
| Elution 3 | Buffer | 60% Ethylene Glycol | pH 4.0 |
|  | SELDI array surfaces | CM10 & Q10 | CM10 |
|  | Average CV (%) | 16.02 | 20.3 |
|  | No. candidate biomarkers | 27 | 20 |
| Elution 4 | Buffer | Organic | 3.0 M Guanidine |
|  | SELDI array surfaces | CM10 | CM10 & IMAC30-Ni |
|  | Average CV (%) | 24.01 | 20.01 |
|  | No. candidate biomarkes | 4 | 28 |

No. of candidate biomarkers are non correlating peaks identified either by ICA or by KW with p < 0.05.
