# Supplementary material for: Plasma proteome analysis of patients with type 1 diabetes with diabetic nephropathy
Source: Proteome Sci. 2010 Feb 3;8:4. doi: 10.1186/1477-5956-8-4 (PMC2827395; doi:10.1186/1477-5956-8-4)
Supplement: Additional file 2 — Supplementary information. Detailed protocols of data acquisition and processing, immunoprecipitation, trypsin digestion and western blotting. [file 1477-5956-8-4-S2.DOC]

**Supplementary information**

**Data Acquisition and Processing**

All of the arrays from both fractionation procedures were read on a PCS4000 Enterprise instrument (Bio-Rad) at two laser settings, which were optimized separately for the lower mass region (2 – 20 kDa) and a higher mass region (4 – 200 kDa). The overlap in optimization ranges ensures an improved detection of peaks in the range 4 – 20 kDa. The matrix attenuation for the low and high mass acquisitions was 1000 Da and 2500 Da, respectively. All spectra were processed using the software supplied with the instrument. Smoothing was performed with a setting of five times the expected peak width and baseline subtracted using default settings. The noise levels were set to 500 Da above the matrix attenuation value to the end of the acquisition range to exclude matrix signals. The spectra were separated by laser setting (low and high) and sample type (clinical samples and reference human plasma). The spectra in each data subset were normalized using the total ion current algorithm included in the instrument software with mass range values identical to the ranges used in the noise calculations and an external coefficient of one. Spectra that had a normalization factor greater than twice the mean normalization factor for a data subset were deleted. A single replicate of each sample was kept in the case where both duplicates were considered outliers unless no appreciable signal was obtained. Peak clusters were selected using the instrument software with a signal-to-noise minimum and valley depth requirement of 5.0 and a minimum threshold of 10%.

**Immunoprecipitation**

An aliquot of Protein G beads were washed with 50 µL of PBS (Gibco, Invitrogen) before incubation with the antibody for 30 min, with shaking at room temperature. The beads added the antibody was washed three times with 50 µL of TBST before incubation with serum for 30 min, shaking, at room temperature. Unbound sample was collected for later analysis and the beads washed three times with 50 µL of PBS. The bound protein was eluted from the beads by adding 15 µL of 0.1% triflouric acid + 50% acetonitrile. As control an identical experiments were carried out simultaneously, but with incubation with PBS instead of an antibody and/or by incubation of PBS instead of plasma.

**Trypsin digestion**

Before digestion with Trypsin Gold (Promega, USA) the protein was precipitated with six volumes of cold (4°C) Acetone and left at -20°C for approximately 4 hours. After decanting the acetone, the protein pellet was dissolved in 10 µL of 6 M guanidine HCl and heated at 95°C for 20 min, followed by dilution with NH_4_HCO_3_. The protein was digested with Trypsin Gold overnight at 37°C. Before the analysis on ESI ion trap MS, the proteins were cleaned with ZipTips® (Millipore, Billerica, Massachusetts, USA).

**Western blots**

Plasma from two DMN, two MIC and two N patients was subjected to SDS-PAGE**,** and the gels where blotted on to nitrocellulose membranes. The membranes were incubated with blocking solution containing anti-apo C1 monoclonal (1:5000 dilution; Abcam, Cambridge, Massachusetts.) antibody and then incubated with blocking solution containing 1:2000 diluted horseradish peroxidise-conjugated secondary antibody. Signals were detected by chemiluminescence using LumiGLO (Cell Signalling, Danvers, Massachusetts, USA). Bands from the Western blotting were scanned and digitized using Fuji LAS3000 (Tokyo, Japan)
